# The invasive and non-invasive monitoring of inspiratory effort and drive during assisted mechanical ventilation

**DOI:** 10.1016/j.aicoj.2025.100003

**Published:** 2026-01-16

**Authors:** Davide Chiumello, Alessandra Muscas, Simone Mancusi, Antonio Fioccola

**Affiliations:** a Department of Health Sciences, University of Milan, Italy; b Department of Anesthesia and Intensive Care, ASST Santi Paolo e Carlo, San Paolo University Hospital Milan, Italy; c Coordinated Research Center on Respiratory Failure, University of Milan, Italy

**Keywords:** ARDS, Assisted mechanical ventilation, Esophageal pressure, Inspiratory effort, PMI, P0.1, ΔPocc, Diaphragm ultrasound

## Abstract

**Background:**

In patients with respiratory failure, assisted mechanical ventilation is often used after the initial acute phase to support patient’s respiratory activity, enhance oxygen exchange, and improve lung ventilation and perfusion. In this phase, monitoring inspiratory effort and respiratory drive might reduce the risk of patient self-inflicted lung injury (P-SILI), thus potentially improving the outcome. P-SILI depends on the negative pressure generated by inspiratory muscles and the positive pressure delivered by the ventilator machine. Measuring the amount of inspiratory pressure generated by the patient and assessing work of breathing is therefore essential. For patients without esophageal or gastric catheters, several non-invasive indices have been developed to estimate inspiratory effort, respiratory drive and evaluate diaphragm function. The aim of this clinical review is to summarize current evidence regarding invasive and non-invasive instruments to monitor inspiratory effort and respiratory drive in patients undergoing assisted mechanical ventilation.

**Main body:**

Esophageal pressure is the reference measure used to evaluate total work of breathing and patient inspiratory effort. Monitoring esophageal swing may help in preventing both over-assistance and under-assistance during mechanical ventilation. The diaphragmatic component of the total work of breathing can be estimated through a gastric catheter, that allows the measurement of transdiaphragmatic pressure. Various non-invasive indices are available in the literature to estimate patients' inspiratory effort, patient’s respiratory drive and diaphragm function. During assisted mechanical ventilation, performing an end-inspiratory pause or an end-expiratory pause allows the measurement of pressure muscle index (PMI), airway occlusion pressure at 100 ms (P0.1), and airway occlusion pressure (ΔPocc). Various cutoffs have been identified in the literature for a low and a high inspiratory effort (PMI, ΔPocc) and respiratory drive (P0.1) during assisted ventilation. Diaphragmatic function can be quickly assessed using ultrasound. Three indices are extensively described in the literature: diaphragm thickness (D_T_), diaphragm excursion (D_E_), and diaphragm thickening fraction (D_Tf_).

**Conclusion:**

Monitoring inspiratory effort and respiratory drive is crucial in current clinical practice to reduce P-SILI. At the present time several invasive and non-invasive tools can quantify inspiratory effort and respiratory drive, and they are well correlated to each other and to the reference measures of patient inspiratory effort. Their use should be encouraged to tailor assisted mechanical ventilation to the individual patient and to promote further studies, that are essential for their ultimate validation.

## 1 Background

Controlled mechanical ventilation is typically employed in the early stages of acute respiratory distress syndrome (ARDS) and acute respiratory failure (ARF) to stabilize gas exchange and mitigate ventilator-induced lung injury (VILI) [[1], [2], [3], [4]]. Subsequently, assisted mechanical ventilation (AMV) is used for its benefits: reduced sedation requirements, improved ventilation and perfusion distribution and enhanced gas exchange [[5], [6], [7]]. In controlled modalities, VILI is primarily caused by the mechanical ventilator through excessive lung stress and strain transmitted to the respiratory system, and via atelectrauma [8]. Nevertheless, assisted modalities can also induce lung damage, known as patient self-inflicted lung injury (P-SILI), where the inspiratory effort generated by respiratory muscles (primarily through diaphragm contraction) is added to the positive pressures generated by the machine. P-SILI may also occur in controlled mechanical ventilation, in patients that are not paralyzed and/or deeply sedated with a spontaneous respiratory activity. Consequently, P-SILI results from the combined effect of the patient's spontaneous effort and the mechanical ventilator [9,10]. P-SILI can occur as weel during spontaneous breathing in patients with severe acute respiratory failure. In this case, a strong inspiratory effort generated by diaphragm contraction can increase lung stress and distension by raising transpulmonary pressure (P_L_) (see Equation 1) [9]. Moreover, prolonged vigorous effort can exacerbate patient distress, complicating the weaning process from mechanical ventilation [11] and potentially having a negative impact on patients’ outcome [[12], [13], [14]]. In AMV modalities, a level of support can be associated to a high inspiratory effort, that can increase the risk of PSILI. Thus, monitoring inspiratory effort in patients undergoing AMV is important. The direct evaluation of negative pressure generated by muscular activity (*i.e* the pleural pressure) should be the reference measure, which is usually estimated through an esophageal catheter [9,15]. Alternatively, several non-invasive indices have been developed over the years to estimate patient inspiratory effort, allowing for accurate assessment and limiting P-SILI, without the need for an esophageal catheter.

In this clinical review, we explore the main invasive and non-invasive tools available to measure inspiratory effort and respiratory drive during AMV with invasive interfaces, with the aim to limit P-SILI.

## 2 Invasive indices of inspiratory effort

### 2.1 Muscle activity, work of breathing, pressure time product and esophageal pressure swing

**Fig. 1:**
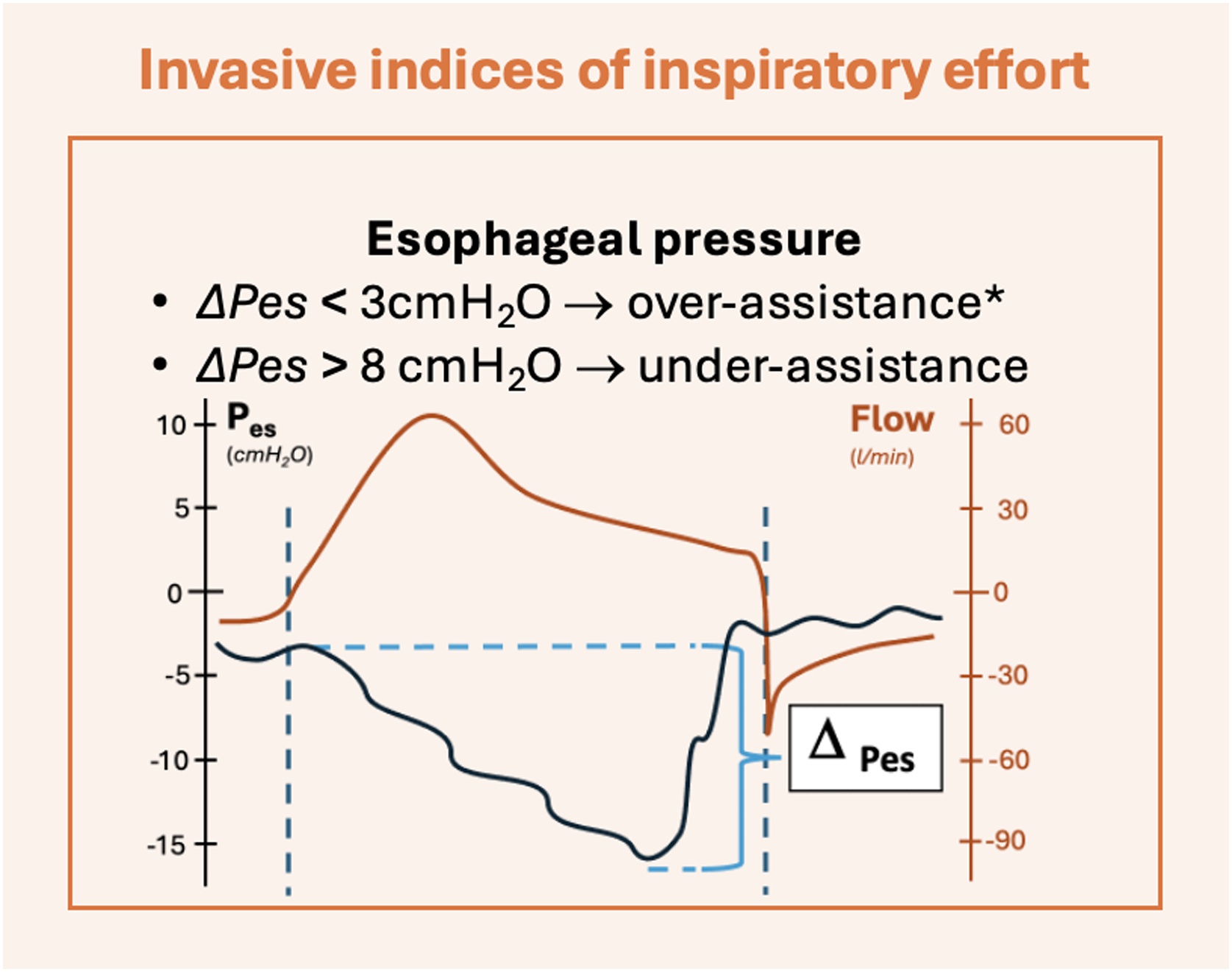
Esophageal pressure. *Legends*: ΔPes, esophageal pressure swing; Pes, esophageal pressure; cmH_2_O, centimeters of water; l, liters; min, minutes. The curves shown in the figures are only representatives. *In the reported example, neuromuscular coupling is intended to be normal, with no patient's muscular weakness.

The esophageal pressure is measured via a catheter placed in the lower third of the esophagus, which should reflect changes in pleural pressure when correctly positioned [[16], [17], [18], [19]]. In controlled mechanical ventilation, in a deeply sedated and/or paralyzed patient, there is a progressive increase in esophageal pressure, peaking at end inspiration (*i.e.*, a positive deflection). Conversely, during AMV, the esophageal pressure records a negative deflection during the inspiratory phase, which measures the intensity of the effort when the patient actively breathes. The moment of higher effort intensity can arrive at different stages, excluded the very early phase of the contraction, *i.e.* the ramp of the inspiratory phase [20] (Fig. 1). The esophageal catheter constitutes an invasive, direct measure of patient’s inspiratory effort, and represents the reference technique with this aim.

The transpulmonary pressure generated during inspiration in patients undergoing mechanical ventilation is the difference of the pressure delivered by the mechanical ventilator in the airways and the negative pleural pressure due to the inspiratory muscle activity, commonly recorded by the esophageal catheter [9] (Fig. 1, equation 1).

$$P_{L}=P_{aw}-\;P_{es}$$

Equation 1. Legends: *P_L_*, transpulmonary pressure; *P_aw_*, airway pressure; *P_es_*, negative esophageal pressure swing.

The reference method to evaluate the inspiratory effort is the global inspiratory muscle pressure (P_mus_). It can be measured by adding the pressure to overcome the elastic recoil of the chest wall (P_cw_) to the absolute value of pleural pressure (P_pl_), for which esophageal pressure (P_es_) constitutes an accurate estimate. The esophageal pressure (P_es_) must be measured at the moment of its maximal change in amplitude (ΔP_es_max_), due to the inspiratory muscle contraction (equation 2, a). Pcw is the product of the chest wall elastance and the tidal volume (equation 2, b).

$${a.\;P}_{mus}=P_{cw}+|\text{Δ}P_{es\_max\;}|$$

$${b.\;P}_{cw}=V_{t}*E_{cw}$$

Equation 2. Legends: $P_{mus}$, global inspiratory muscle pressure; |ΔP_es_max_|, absolute value of esophageal pressure swing at the moment of its maximal change in amplitude; $P_{cw}$, pressure to overcome the elastic recoil of the chest wall. Vt, tidal volume; E_cw_, chest wall elastance.

The most complete measure of the total energy dissipated by respiratory muscles during the inspiratory phase is the work of breathing (WOB). WOB can be calculated as the product of the pressure generated by respiratory muscles and the pulmonary volumes obtained. Therefore, WOB can be expressed as the integral of global inspiratory muscle pressure per volume in time. It corresponds to the area of a pressure volume diagram during mechanical ventilation (equation 3) [21].

$$WOB=\int_{to}^{T1}P_{mus}*V*dt$$

Equation 3. Legends: *WOB*, work of breathing; *t0*, time at the start of the inspiratory phase; *T1*, time at the end of the inspiratory phase; *P_mus_*, global inspiratory muscle pressure; *V*, tidal volume; *dt*, time differential.

Another way to evaluate the energy dissipated by respiratory muscles is the pressure time product of the esophageal pressure (PTP_es_). PTP_es_ is calculated as the integral of the product of the global inspiratory muscle pressure and time of muscle contraction during the inspiratory phase. Even if their physiological meaning is similar, WOB estimates the inspiratory effort as the integral of pressure over volume, whilst PTP_es_ is the integral of pressure over time (equation 4) [21,22]. For these feature, PTP_es_ is also accountable in case of isometric contraction, thus being more reliable than WOB in this situation [20].

$${PTP}_{es}=\int_{to}^{T1}P_{mus}*ti*dt$$

Equation 4. Legends: *PTP_es_*, esophageal pressure time product; *t0*, time at the start of the inspiratory phase; *T1*, time at the end of the inspiratory phase*; P_mus_*, global inspiratory muscle pressure; *ti, inspiratory time; dt, time differential.*

It can be difficult to obtain a direct calculation of WOB and PTP_es_ in daily clinical practice. The difference between the esophageal pressure at the start of the inspiratory phase and its most negative value, also known as esophageal swing (ΔP_es_) is widely used as an easy to measure surrogate of WOB and PTP_es_ (equation 5) [23].

$${\text{Δ}P}_{es}=P_{es}\left(t_{0}\right)-\;P_{es}\left(T_{min}\right)$$

Equation 5. Legends: Δ*P_es_*, esophageal swing; *P_es_*, esophageal pressure; *t_0_*, start of the inspiratory phase*; T_min_,* time of the inspiratory phase when the P_es_ reaches its most negative value.

Esophageal swing has an overall good correlation with the PTP_es_ of patients undergoing assisted ventilation [23]. In patients with low chest wall elastance (with a low P_cw_), values of ΔP_es_ between 3 and 8 cmH_2_O can be reasonably aligned to values of P_mus_ of 5–10 cmH2O [24]. It has been shown how, with usual values of inspiratory time and respiratory rate, these levels of P_mus_ can reasonably correspond with PTP_es_ values between 50 and 150 cmH_2_O*s*min^−1^, identified as reasonable target range of patient inspiratory effort [25].

These considerations do not apply to certain categories of patients, in which chest wall elastance is higher and P_*cw*_ is not negligible, as it happens in earlier stages of ARDS [26].

### 2.2 Transdiaphragmatic pressure

**Fig. 2:**
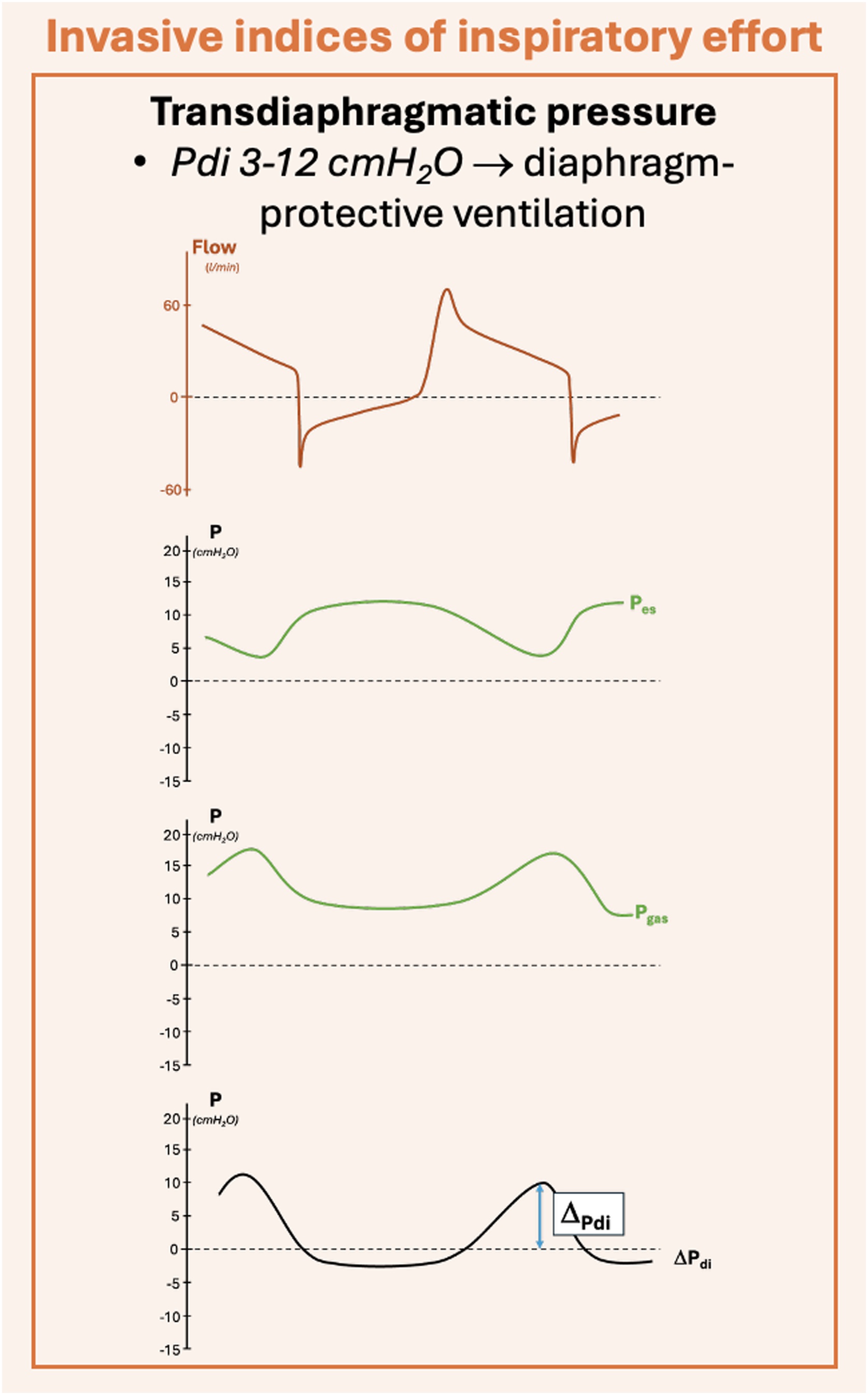
Transdiaphragmatic pressure. *Legends*: P, pressure; ΔPdi, transdiaphragmatic pressure; l, liters; min, minutes; cmH_2_O, centimeters of water; Pes, esophageal pressure; Pga, gastric pressure. The curves shown in the figure are only representatives.

Beyond the role of esophageal pressure in measuring excessive or insufficient muscular effort during AMV, there is growing interest in diaphragm function and its impairment due to muscle inactivity and unloading [[28], [29], [30], [31], [32]]. The transdiaphragmatic pressure is computed as the difference between gastric and esophageal pressures, and is the reference technique to measure the diaphragmatic component of the work of breathing [24] (Fig. 2, Equation 5).

$$\text{Δ}P_{di}=P_{ga}-P_{es}$$

Equation 5. Legends: Δ P_di_, transdiaphragmatic pressure; P_ga_, gastric pressure; P_es_, absolute numerical value of esophageal pressure.

Accordingly, a new concept of lung-diaphragm protective mechanical ventilation has been suggested to maintain diaphragm activity within the limits of physiological efforts [27].

De Vries *et al.* evaluated the possibility to titrate inspiratory support to achieve a transdiaphragmatic pressure between 3 and 12 cmH_2_O, comparing this to standard lung protective ventilation [33]. In Table 1 we report the main advantages and disadvantages of monitoring inspiratory effort with an esophageal and/or a gastric catheter.

**Table 1:** Pros and cons of esophageal and transdiaphragmatic pressure measurement during AMV.

	Pros	Cons
Esophageal pressure	Gold standard for measuring patient inspiratory effort [23,25].	Invasive device: - May need repositioning [18]; - May cause patient discomfort; - Increases costs; - Needs dedicated equipment; - Needs dedicated expertise [9].
Transdiaphragmatic pressure	Allows precise evaluation of diaphragmatic effort, aiding in the improvement of diaphragmatic function during AMV and possibly preventing VIDD [34].	A gastric balloon and/or an additional catheter is required together with the esophageal one, potentially increasing the risk of displacement, patient discomfort, and costs [25].

AMV, assisted mechanical ventilation; VIDD, ventilator induced diaphragm dysfunction.

## 3 Non-invasive indices of inspiratory effort

Several non-invasive indices have been developed to measure inspiratory effort in patients undergoing AMV based on airway pressure and flow curves displayed on the mechanical ventilator.

### 3.1 Pressure Muscle Index (PMI)

**Fig. 3:**
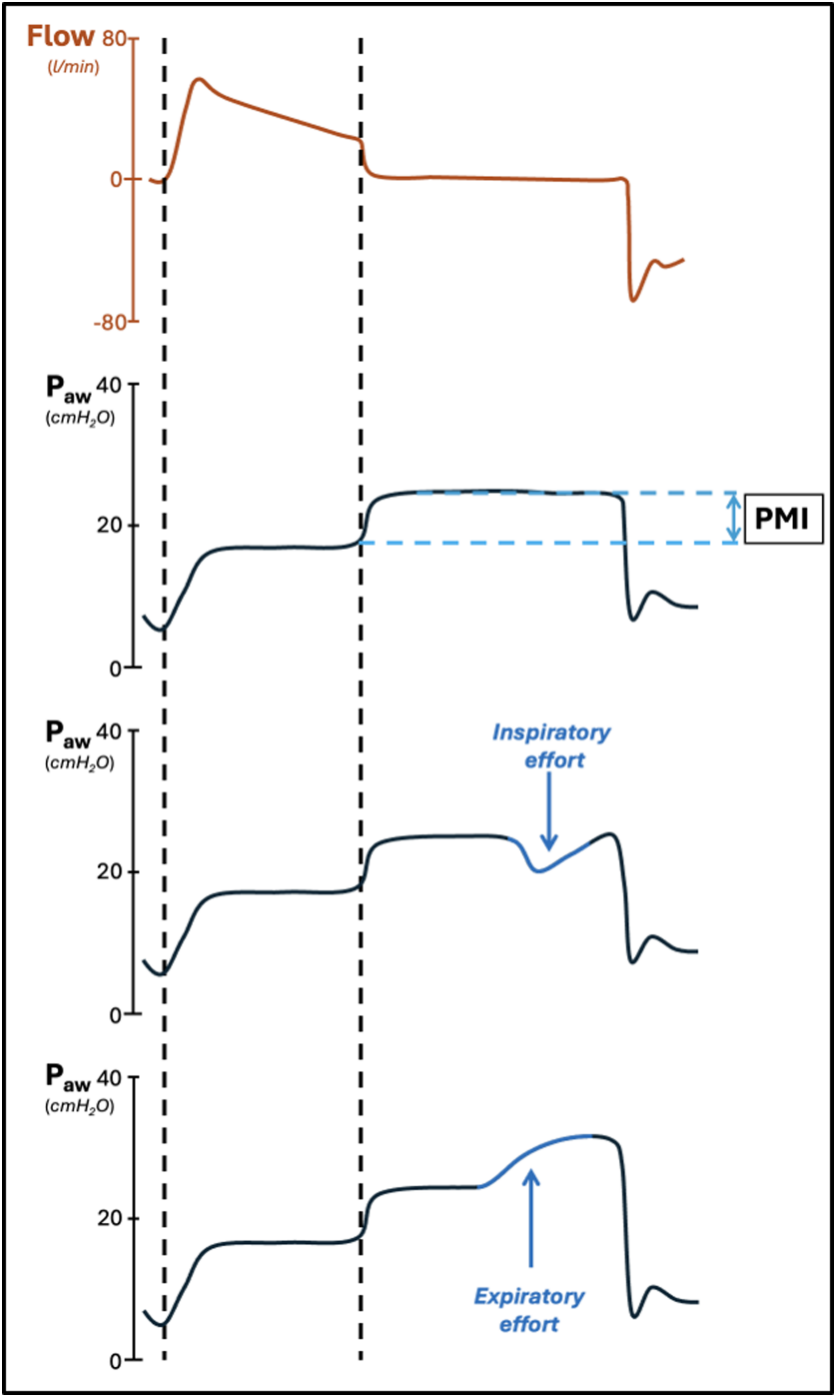
Influence of an expiratory or inspiratory patient effort during the inspiratory hold. *Legends*: P_aw_, airway pressure; cmH_2_O, centimeters of water; PMI, pressure muscle index; l, liters; min, minute. Dashed vertical lines: start and end of the inspiratory phase. The curves shown in the figures are only representatives.

The first description of an end-inspiratory pause to estimate the patients’ inspiratory effort in those ventilated with pressure support ventilation dates back to 1997 [35]. In this paper, the authors described the pressure muscle index (PMI) as the difference between the end-inspiratory occlusion plateau pressure and the airway pressure before the occlusion (i.e., PEEP + pressure support) (Fig. 3). During an inspiratory hold, after a variable amount of time, the inspiratory effort of the patient ceases and the difference in pressure between the two mentioned points (PMI) can be used to estimate the contribution of patient’s inspiratory effort to the pressure wave. This phenomenon is due to the relaxation of respiratory muscles of the patient, that leads to an increase in airway pressure [35].

PMI is well correlated with the pressure developed by inspiratory muscles and the pressure-time product per breath (PTP_es_/b) and per minute (PTP_es_/m) [35]. These data corroborate the validity of PMI as a predictor of inspiratory effort. A cutoff of 6 cmH_2_O for PMI has been identified as a marker of under-assistance, being associated with a PTP_es_/m greater than 125 cmH_2_O*s/min with high sensitivity and specificity (0.89; 0.89) [35]. More recently, in a study on 18 patients undergoing pressure support ventilation, Natalini *et al.* found that a cutoff of 1 cmH_2_O for PMI was able to identify over-assistance [37]. It is important to underline that the authors showed an excellent negative predictive value for identifying over-assistance (0.96; 95% CI 0.91−0.99), although with a low positive predictive value (0.33; 95% CI 0.23 – 0.45). Thus, a PMI < 1 cmH_2_O was able to accurately predict over-assistance, even if a PMI > 1 cmH_2_O could not rule it out. A recently published prospective physiological study confirms these findings, validating the high negative predictive value of PMI for identifying both under- and over-assistance, while still emphasizing its low positive predictive value [38]. It is important to remember that an unstable airway plateau pressure can negatively impact the computation of the pressure muscle index (see Table 2 and Fig. 3). The incidence of this artifact has been found to be high in retrospective analyses [39,40]. Bianchi e*t al.* proposed three easy-to-assess criteria to determine the reliability of the airway plateau pressure: a rapid reach of the plateau pressure, in less than 800 ms; an adequate duration of the plateau (> 2 s); and low variability during the observation (<0.6 cmH_2_O/s) [41]. The morphological analysis of the curve may allow clinicians to identify typical artifacts. Among these, a negative PMI may indicate the presence of an air leak during the inspiratory pause. In case of further inspiratory effort, a sudden drop in the plateau pressure can be observed: in this case, the PMI should be measured immediately before the drop, in order to avoid an underestimation of patient’s effort (see Fig. 3). Finally, an expiratory effort may be detected by observing a progressive increase in the airway plateau pressure during the pause [42] (see Fig. 3). In a recent analysis published by Docci *et al.* [43], the inverse relation between the level of ventilator support and PMI has further highlighted the role of this index in identifying the appropriate level of assistance: the authors found out increasing levels of PMI in patients receiving lower levels of pressure support [43]. It is important to remind that PMI is measured at zero flow, using the plateau airway pressure (P_awplat_). Thus, it is not sensitive to the effort that the patient uses to overcome airway resistances. This is a potential limit for PMI to evaluate inspiratory effort in patients with longer airway circuits and/or in patients with bronchoconstriction [35].

**Table 2:** Pros and Cons of pressure and flow curve analysis during AMV to predict patient’s inspiratory effort and drive during assisted mechanical ventilation.

	Pros	Cons
PMI; P0.1; ΔP_occ_	Non-invasive and easy to assess with an end-inspiratory (PMI) or an end-expiratory (P0.1; ΔP_occ_) pause; These indices are well correlated with ΔP_es_ and P_mus_ [36,46,50].	- PMI has a low positive predictive value in identifying under- and over-assistance [38]; - PMI cannot be measured when inspiratory or expiratory efforts occur early during the pause, thus not allowing a stability of the plateau for at least 2 seconds [41]; - Circuit air leaks, longer airway circuits and the presence of bronchoconstriction may negatively influence the accuracy of all these parameters, that are measured during an inspiratory/expiratory hold, at zero flow conditions [35]. - P0.1 is a more reliable index of inspiratory drive, rather than inspiratory effort [59,60]; - The potential difference between the value obtained by a 100 ms occlusion and the value obtained by extrapolation (P0.1vent) during automated measurement might be an additional limit [66].
Flow index	- Non-invasive and easy to assess [49]; - Allows continuous evaluation over time, without the need for a ventilation pause [49].	- Requires algorithmic analysis to be measured [47]; - The algorithm has not yet been widely implemented on ICU mechanical ventilators [47].

PMI, pressure muscle index; P0.1, airway occlusion pressure at 100 ms; ΔPocc, airway occlusion pressure; P0.1, airway occlusion pressure at 100 ms directly measured by the mechanical ventilator; P_es_, esophageal pressure; P_mus_, global inspiratory muscle pressure; AMV, assisted mechanical ventilation; ICU, intensive care unit.

### 3.2 Airway occlusion pressure (ΔP_occ_)

Airway occlusion pressure represents the maximal negative swing during an end-expiratory pause on the airway pressure curve, in correspondence with the patient’s inspiratory effort. It has similar sensitivity and specificity when compared to PMI [42,44] and is superior to P0.1 in identifying low and high inspiratory effort, as demonstrated by stronger association (higher R^2^ and AUC) with esophageal and transdiaphragmatic pressures’ values in mechanically ventilated patients [44,45]. This feature may depend from the fact that P0.1 only regards the first part of the deflection during the inspiratory phase, thus making it a more reliable index of inspiratory drive, rather than global inspiratory effort [44].

There are no definite safe cutoffs to determine low and high effort using ΔP_occ_ in the literature [40,44,45]. In a study on 246 patients undergoing pressure support ventilation, the authors compared levels of P0.1 and ΔP_occ_ with P_mus_ and the pressure-time product of the global inspiratory muscle pressure per minute (PTP_mus_/min). In this investigation, the authors defined a low inspiratory effort as a P_mus_ and PTP_mus_/min respectively lower than 5 cmH_2_O and 50 cmH_2_O*s*min^−1^. At the opposite, they defined a high inspiratory effort as a P_mus_ or PTP_mus_/min respectively higher than 10 cmH_2_O and 200 cmH_2_O*s*min^−1^. In this way they identified a cutoff of about 1 and 6 cmH2O respectively for P0.1 and ΔP_occ_ to identify low inspiratory drive and effort, whilst a cutoff of about 2 and 9 cmH_2_O were identified to identify a high inspiratory drive and effort using P0.1 and ΔP_occ_, respectively [42]. In another analysis on 28 patients undergoing assisted mechanical ventilation, using cross-validation, the authors found a conversion ratio potentially able to derive P_mus_ and P_es_ from ΔP_occ_ (P_mus_/ΔP_occ_ = -0.74, 95% CI: −0.69, −0.78; ΔP_es_/ΔP_occ_ = 0.66, 95% CI: 0.61–0.70). These ratios showed a high AUC (0.94) when validated on an external study population [45]. ΔP_occ_ accurately detected a P_mus_ higher than 10 cmH_2_O and a transpulmonary pressure higher than 15 cmH_2_O [45]. Adopting these conversion ratios and using safe limits of 3–8 cmH_2_O for ΔP_es_ and 5–10 cmH_2_O for Pmus, a possible range for ΔP_occ_ to identify low and high inspiratory effort might be between -13 and -6 cmH_2_O. For sake of simplicity, we may adopt the absolute values of ΔP_occ_, and identify safe values of 6–13 cmH_2_O to rule out over and under-assistance (see Fig. 5).

### 3.3 Inspiratory flow index

**Fig. 4:**
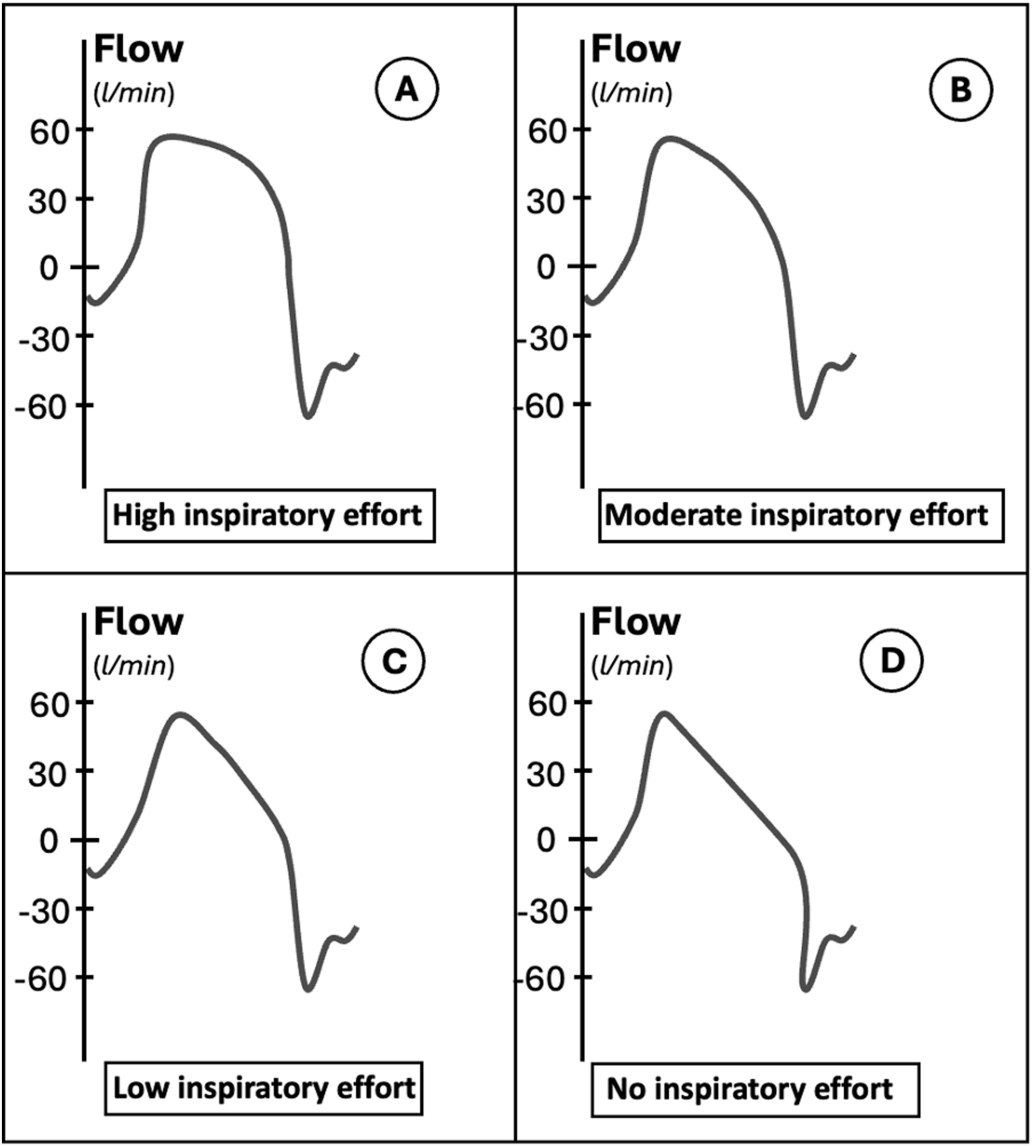
Flow index in patients with high, moderate and low inspiratory effort. A progressive decrease of the flow curve upward convexity can be seen from patients with high inspiratory effort (panel A) to patients with moderate and low inspiratory effort (panel B and C). In panel D the shape of a patient with no inspiratory effort is shown. These curves are only representatives. *Legends*: l, liters; min, minute.

**Fig. 5:**
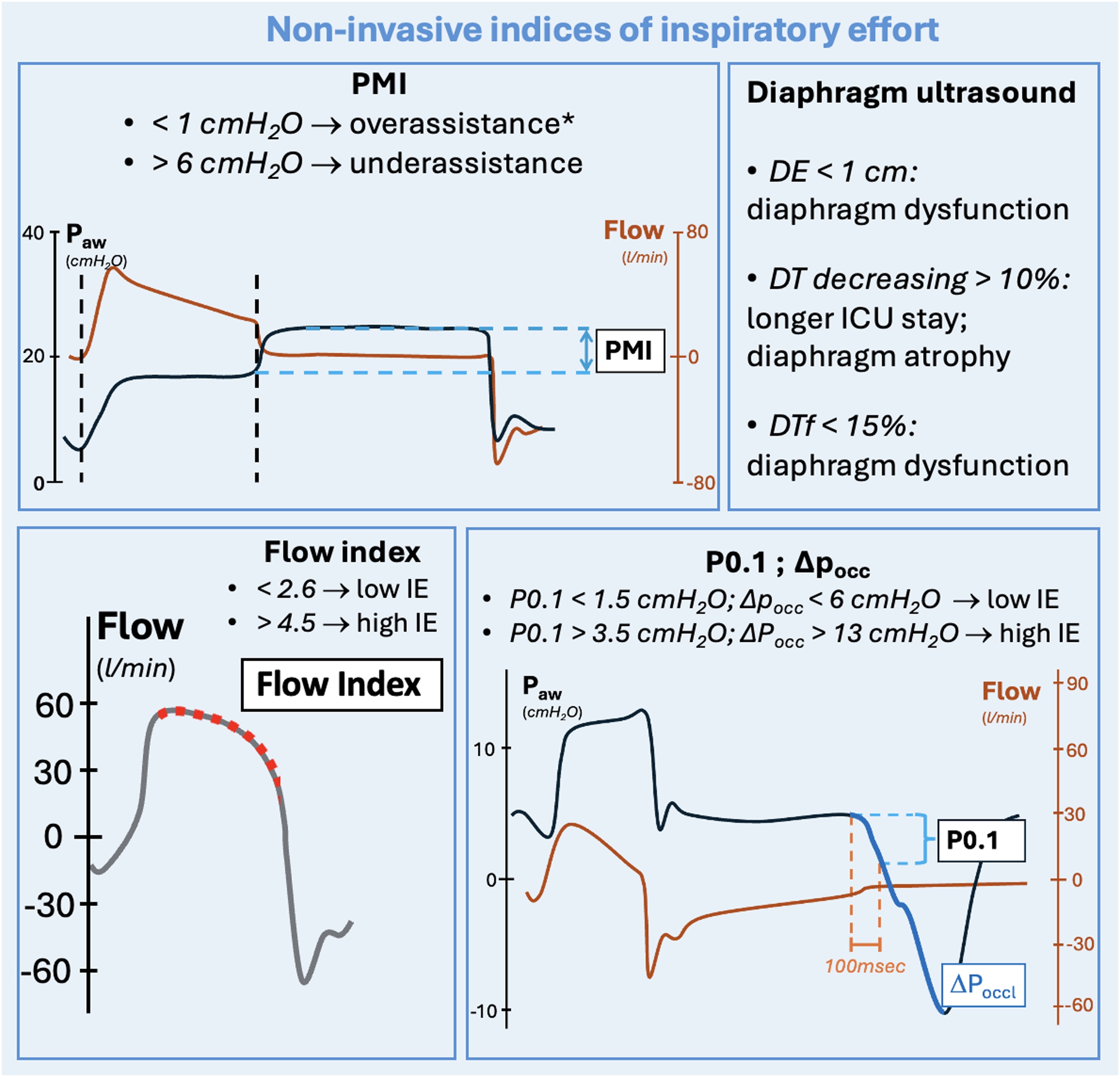
Non-invasive indices to estimate patient’s inspiratory effort and drive during mechanical ventilation. *Legends*: PMI, pressure muscle index; P_aw_, airway pressure; cmH_2_O, centimeters of water; l, liters; min, minutes; DE, diaphragm excursion; DT, diaphragm thickening; DTf, diaphragm thickening fraction; IE, inspiratory effort; P0.1, airway occlusion pressure at 100 ms; ΔP_occ,_ airway occlusion pressure. The curves shown in the figure are only representatives. *In the reported example, neuromuscular coupling is intended to be normal, and the patient is not intended to experience muscular weakness.

The morphological analysis of the inspiratory flow curve during pressure support ventilation from the end of the ramp (when the target inspiratory pressure is reached) to the expiratory trigger is also known as flow index (see Fig. 5). It may be informative about patients’ inspiratory effort. In a passively ventilated patient, in pressure mode, the drop of the flow curve is linear. This drop depends on two factors: the difference between the pressure delivered by the ventilator and the alveolar pressure and the respiratory mechanics. This difference is maximal at the beginning of the inspiration and minimal at the expiratory trigger. In presence of the patient’s inspiratory effort, the drop is not linear, and a downward concavity can be observed in the flow curve [45]. This is because P_mus_ is added to the P_aw_ generated by the ventilator, thus inducing a higher flow in the initial part of the inspiratory phase (see Fig. 4). The drop of the flow curve during pressure support ventilation can be described by the following equation (Equation 6):

$$\Phi =a+b*{\Delta time}^{c}$$

Equation 6. Legends: *Φ*, instantaneous flow; *a*, peak inspiratory flow immediately after the ramp; *b*, rate of flow reduction; *c*, downward concavity of the flow trace after the inspiratory ramp, also indicated as *flow index*.

Where *Φ* represents the instantaneous flow, *a* is the peak inspiratory flow immediately after the ramp, *b* is the rate of flow reduction, and *c* describes the downward concavity displayed on the trace, *i.e.* the flow index [47]. The parameter *c* can be determined via a mathematical curve analysis. It is equal to 1 when the flow trace is linear during the inspiratory phase, indicating the absence of patient’s inspiratory effort. It gradually increases in case of greater downward concavities, thus reflecting higher patient effort. Differently from other non-invasive parameters of inspiratory effort, the flow index is insensitive to intrinsic PEEP, thus being in theory only dependent from effort during the inspiratory phase. This feature depends from the fact that *Φ* is measured on the flow trace and not on pressure waves, as it happens with PMI, P_0.1_ and ΔP_occ_. A linear relationship between the increases in the flow index and invasive indexes of patient effort (P_mus_ and PTP_es_) was recently described [47], thereby strengthening the rationale for its clinical use. In the linear mixed model used for the analysis, the authors demonstrated the independent association of the flow index with patient effort, showing that a unitary increase in the flow index corresponded to an increase of 0.33 cmH_2_O of P_mus_, and was also strongly correlated with PTP_es_ [47]. Two cut-offs of the flow index to predict the amount of inspiratory effort during assisted ventilation have been determined, using P_mus_ as the reference method in patients equipped with an esophageal catheter: a flow index < 2.6 predicted low inspiratory effort, whilst a flow index > 4.5 was associated with high inspiratory effort. The low cut-off predicted a P_mus_ lower than 5 cmH_2_O with an AUC of 0.89, whilst the high cut-off predicted a P_mus_ higher than 10 cmH_2_O with an AUC of 0.80 [48]. A similar cut-off to identify over-assistance was found in a recent analysis, thus reinforcing previous results [49]. Unfortunately, flow index is not directly measurable by the clinician and it needs the algorithmic analysis of the curve. In Table 2, we synthetize the main advantages and disadvantages of using pressure and flow curve analysis derived indexes to evaluate patient’s inspiratory effort during AMV.

## 4 Ultrasound indexes to estimate diaphragm function

Ultrasound is an increasingly recognised tool to non-invasively estimate diaphragm function and to correct levels of assistance in patients undergoing AMV [50].

The main ultrasound parameters to evaluate diaphragm function are diaphragm excursion (D_E_), diaphragm thickness (D_T_), and diaphragm thickening fraction (D_Tf_). A recent consensus established that, in patients undergoing AMV, a unilateral ultrasound assessment of the right hemidiaphragm is acceptable for all these parameters, unless unilateral dysfunction is suspected [26]. Diaphragm excursion during inspiratory effort can be measured with the patient in a supine position, using an ultrasound probe placed over one of the lower intercostal spaces in the right anterior or left anterior axillary line, for the assessment of right or left hemidiaphragm. D_E_ is defined as the maximum amplitude of diaphragm movement recorded in M-mode during inspiration [51]. A D_E_ of less than 1 cm during a spontaneous breathing trial has been used to define diaphragm dysfunction in patients undergoing mechanical ventilation [51].

Diaphragm thickness can be similarly measured during the inspiratory phase. In a study conducted on more than 200 mechanically ventilated patients, a decrease over time of diaphragm thickness of more than 10 percent has been associated with prolonged duration of mechanical ventilation and longer intensive care unit (ICU) stay [34]. In this analysis, D_T_ was measured every day during ICU stay. The D_T_ value on the day of extubation or after one week from the beginning of mechanical ventilation was compared to the value measured at the admission time [34]. The association between the use of sedatives and D_T_ has also been questioned: in a randomized controlled trial (RCT) on 66 patients undergoing spontaneous ventilation, the authors showed how withholding sedatives immediately increased diaphragm thickness [52].

Diaphragm thickening fraction is another important non-invasive index of diaphragm function that has been extensively studied in the literature. Its values in healthy, spontaneously breathing patients during a deep inspiration at total lung capacity may vary from 50 to 200 percent [53] (equation 7).

$${\text{D}}_{\text{T}\text{f}}=\;\frac{{\text{D}}_{\text{T}\text{i}\text{n}\text{s}\text{p}}-{\text{D}}_{\text{T}\text{e}\text{x}\text{p}}}{{\text{D}}_{\text{T}\text{e}\text{x}\text{p}}}\;$$

Equation 7. Legends: *D_Tf_*, diaphragm thickening fraction; *D_Tinsp_*, diaphragm thickening at end-inspiration; *D_Texp_*, diaphragm thickness at end-expiration.

A D_Tf_ of more than 25% in patients ventilated in pressure support mode was correlated with simpler weaning from mechanical ventilation with a high AUC (0.9) [54]. A lower cut-off of 15% at a single measure during the first 48 h of mechanical ventilation was a possible good predictor of diaphragm dysfunction [55]. A wider range of 15–30 percent of D_Tf_ was associated with stable muscle thickness over time and with a shorter duration of mechanical ventilation [34]. D_Tf_ was also directly associated with the total work of breathing measured as PTP_es_ per breath and per minute, thus correlating this non-invasive index with a reference measure for measuring patient inspiratory effort [23]. However, its correlation with the reference measure of inspiratory effort was weak (R^2^ = 0.326), being higher after excluding patients with diaphragmatic dysfunction from the analysis (R^2^ = 0.889) [23].

Despite the validation and interchangeable clinical use of D_E_, D_T_, and D_Tf_, two recent meta-analyses showed that measuring D_Tf_ is superior to measuring D_E_ and D_T_ in predicting both success and failure of weaning from mechanical ventilation [56,57].

Being D_E_, D_T_, and D_Tf_ three ultrasound derived indices, it is important to bear in mind that intra-operator variability can make them less accurate [50]. When employing these indices, it is important to ensure adequate formation for the operators involved [58].

In Table 3 we synthetize the main advantages of using ultrasound-derived indexes to evaluate diaphragm function, whilst in Fig. 5 we summarize safe cutoffs identified in the literature for all non-invasive indices of inspiratory effort and respiratory drive.

**Table 3:** Pros and cons of ultrasound-derived indexes to evaluate diaphragm function.

	Pros	Cons
D_T_, D_E_, D_Tf_	Non-invasive measures; Unilateral evaluation is adequate unless a unilateral deficit is suspected [24].	Do not allow continuous measurements [57,58,62]; Operator-dependent [26] [50]
D_Tf_ has been shown to be superior to D_T_ and D_E_ in estimating patient’s diaphragmatic function.		

D_T_, diaphragm thickness; D_E_, diaphragm excursion; D_Tf_, diaphragm thickening fraction.

## 5 Non-invasive indices of respiratory drive

### 5.1 Airway occlusion pressure at 100 ms (P0.1)

The respiratory drive is the intensity of the neural stimulus to breath, which can be excessively low or high in critically ill patients, possibly resulting in a too low or too high inspiratory effort. Airway occlusion pressure at 100 ms represents the drop in airway pressure 100 ms after the onset of a spontaneous inspiratory act, during an end-expiratory pause (Fig. 4). No flow is present when P0.1 is measured, and lung volume equals the end-expiratory lung volume during the measurement. Thanks to these two features, P0.1 is ideally unaffected by resistive components and by patient’s respiratory mechanics [59], thus constituting a valid index of patient’s respiratory drive [60]. However, it has recently been shown how the measure of P0.1 is impaired in case of high airway resistances [61]. An increased P0.1 reflects a strong inspiratory effort only when neuromuscular coupling is preserved (*i.e.*, in the absence of significant muscle weakness), being not an adequate index of inspiratory effort in all patients [62].

Established this argument, P0.1 has often been compared in the literature with indexes of inspiratory effort, with varying results. In a prospective, randomized, crossover physiological trial on 14 spontaneously breathing mechanically ventilated patients, the authors showed that a P0.1 higher than 3.5 cmH_2_O had high sensitivity and specificity (92% and 89%, respectively) for detecting a PTP_es_ higher than 200 cmH_2_O*s/min, being a possible cutoff to identify under-assistance during assisted mechanical ventilation [63]. The high cutoff of 3.5 cmH_2_O has been further validated by Le Marec *et al.* [64]. In this multicenter study, the authors showed that patients with a P0.1 higher than 3.5 cmH_2_O before extubation were more likely to experience dyspnoea during spontaneous breathing (see Fig. 5). Higher 90-day mortality was also found in patients with a P0.1 out of range. On the other hand, Pletsch-Assuncao *et al.* identified a P0.1 value lower than 1.5 cmH_2_O as correlated to over-assistance, associated with a PTP_es_ of less than 50 cmH_2_O*s/min [65] (see Fig. 5). Similar high cutoffs have been validated by other investigators [25,65]. Modern ventilators have the capability to automatically measure P0.1 (P0.1_vent_). In a recent study on five ventilators in pressure support mode, the authors showed good correlation between relative changes of P0.1_vent_ and P0.1 measured with an offline analysis of the airway pressure curve [66]. The good correlation of P0.1_vent_ when compared to the reference P0.1 has been further demonstrated by Telias *et al.*, using different mechanical ventilators [59]. P0.1_vent_ constitutes, similarly to P0.1, an indicator of patient’s respiratory drive. Nevertheless, it has also demonstrated a good correlation with the reference measure of inspiratory work, PTP_es_, with an area under the curve (AUC) of 0.81 and 0.92 for detecting high and low patient effort, respectively. P0.1_vent_ is also linked with the electrical activity of the diaphragm (Ea_di_) in healthy volunteers and patients [59], thus highlighting its role in evaluating respiratory drive activity. P0.1_vent_ must be interpreted with caution. In a comparative analysis between P0.1_vent_ and P0.1 following an offline evaluation, despite a generally strong correlation, P0.1_vent_ was, on average, higher than the reference measurement (mean bias: 1.3 cmH_2_O) [59]. Higher differences, with potential increased impact on the accuracy of the measure, were observed in patients ventilated with longer ventilation circuits [67]. As also underlined in Table 2, This phenomenon might bring the clinician to overestimate patients’ inspiratory drive when considering P0.1_vent_ (see Table 2).

## 6 Absolute values of ΔP_occ_ and P0.1

For sake of simplicity, we adopted absolute values when talking about safe cut-offs of ΔP_occ_ and P0.1. Even if this is not physiologically correct, being these two measures expression of a negative deflection on the airway pressure curve, this aligns with the tendence of the most recent literature on the topic, and helps the reader to more easily interpret their values.

## 7 Conclusions

During assisted and controlled mechanical ventilation in patients that are not deeply sedated and/or paralyzed, the presence of inspiratory muscle activity can promote P-SILI, which is directly associated with the inspiratory effort generated by respiratory muscles. The respiratory drive can promote a too high or low inspiratory effort. Inspiratory drive and effort should then be measured. The assessment of both invasive and non-invasive measures of spontaneous respiratory activity and respiratory drive can improve understanding of the patient’s condition and clinical course, allowing for personalised mechanical ventilation [68]. Esophageal and transdiaphragmatic pressure remain the two reference techniques to evaluate the whole inspiratory muscle activity and the diaphragmatic component. Several non-invasive indices may replace them, especially in contexts where esophageal/gastric catheters are not available. Although optimal and safe targets of spontaneous inspiratory activity are not yet well defined and further studies are needed for their definition, several data show that measurement of these indexes might help the clinician, thus avoiding under and overassistance during assisted mechanical ventilation.

## CRediT authorship contribution statement

Professor Davide Chiumello has a long-lasting clinical and research activity on mechanical ventilation and lung pathologies treated in the intensive care unit; Doctor Antonio Fioccola is an Anesthetist and Intensive Care Physician working at San Paolo University Hospital. He performed clinical and preclinical research on acute respiratory distress syndrome and acute kidney injury; Doctor Alessandra Muscas and Doctor Simone Mancusi are two Anesthesia and Intensive Care Unit Residents working at San Paolo University Hospital.

DC, AM and AF wrote the text, SM produced the figures, DC and AF revised the text.

## Consent for publication

Not applicable

## Ethics approval and consent to participate

Not applicable

## Funding

No funding has been placed for this study

## Availability of data and material

Not applicable

## Declaration of competing interest

The authors declare that they have no competing interests

## References

[1] Chiumello D., Fioccola A. (2024 Dec 1). Recent advances in cardiorespiratory monitoring in acute respiratory distress syndrome patients. J Intensive Care [Internet].

[2] Chiumello D., Carlesso E., Cadringher P., Caironi P., Valenza F., Polli F. (2008 Aug 15). Lung stress and strain during mechanical ventilation for acute respiratory distress syndrome. Am J Respir Crit Care Med [Internet].

[3] Chiumello D., Brochard L., Marini J.J., Slutsky A.S., Mancebo J., Ranieri V.M. (2017 Sep 12). Respiratory support in patients with acute respiratory distress syndrome: an expert opinion. Crit Care [Internet].

[4] Gattinoni L., Tonetti T., Cressoni M., Cadringher P., Herrmann P., Moerer O. (2016 Oct 1). Ventilator-related causes of lung injury: the mechanical power. Intensive Care Med [Internet].

[5] Spieth P.M., Carvalho A.R., Güldner A., Kasper M., Schubert R., Carvalho N.C. (2011). Pressure support improves oxygenation and lung protection compared to pressure-controlled ventilation and is further improved by random variation of pressure support. Crit Care Med [Internet].

[6] Blankman P., Hasan D., Van Mourik M.S., Gommers D. (2013 Jun). Ventilation distribution measured with EIT at varying levels of pressure support and Neurally Adjusted Ventilatory Assist in patients with ALI. Intensive Care Med [Internet].

[7] Dries D.J. (2016). Assisted ventilation. J Burn Care Res [Internet].

[8] Gattinoni L., Quintel M., Marini J.J. (2018 Oct 25). Volutrauma and atelectrauma: which is worse?. Crit Care [Internet].

[9] Gattinoni L., Giosa L., Bonifazi M., Pasticci I., Busana M., Macri M. (2019 Aug 3). Targeting transpulmonary pressure to prevent ventilator-induced lung injury. Expert Rev Respir Med..

[10] Yoshida T., Grieco D.L., Brochard L., Fujino Y. (2020 Feb 1). Patient self-inflicted lung injury and positive end-expiratory pressure for safe spontaneous breathing. Curr Opin Crit Care [Internet].

[11] Jubran A., Grant B.J.B., Laghi F., Parthasarathy S., Tobin M.J. (2005 Jun 1). Weaning prediction: esophageal pressure monitoring complements readiness testing. Am J Respir Crit Care Med [Internet].

[12] Esnault P., Cardinale M., Hraiech S., Goutorbe P., Baumstrack K., Prud’homme E. (2020 Oct 15). High respiratory drive and excessive respiratory efforts predict relapse of respiratory failure in critically ill patients with COVID-19. Am J Respir Crit Care Med [Internet].

[13] Parrilla-Gómez F.J., Castellví-Font A., Boutonnet V., Parrilla-Gómez A., Antolín Terreros M., Mestre Somoza C. (2025 Oct 1). Association of breathing effort with survival in patients with acute respiratory distress syndrome. Crit Care Med..

[14] Grassi A., Bianchi I., Teggia Droghi M., Miori S., Bruno I., Balzani E. (2025 Nov). Increased driving pressure during assisted ventilation for hypoxemic respiratory failure is associated with lower ICU survival: the ICEBERG study. Am J Respir Crit Care Med..

[15] Jonkman A.H., Telias I., Spinelli E., Akoumianaki E., Piquilloud L. (2023 Jun 30). The oesophageal balloon for respiratory monitoring in ventilated patients: updated clinical review and practical aspects. Eur Respir Rev [Internet].

[16] Chiumello D., Consonni D., Coppola S., Froio S., Crimella F., Colombo A. (2016 Dec 1). The occlusion tests and end-expiratory esophageal pressure: measurements and comparison in controlled and assisted ventilation. Ann Intensive Care [Internet].

[17] Akoumianaki E., Maggiore S.M., Valenza F., Bellani G., Jubran A., Loring S.H. (2014 Mar 1). The application of esophageal pressure measurement in patients with respiratory failure. Am J Respir Crit Care Med..

[18] Baydur A., Behrakis P.K., Zin W.A., Jaeger M., Milic-Emili J. (1982). A simple method for assessing the validity of the esophageal balloon technique. Am Rev Respir Dis [Internet].

[19] Abbate G., Colombo S.M., Semenzin C., Sato N., Liu K., Ainola C. (2024 Dec 1). Comparative analysis of novel esophageal pressure monitoring catheters versus commercially available alternatives in a biomechanical model of the thoracic cavity. Sci Rep [Internet].

[20] Mauri T., Yoshida T., Bellani G., Goligher E.C., Carteaux G., Rittayamai N. (2016 Sep 1). Esophageal and transpulmonary pressure in the clinical setting: meaning, usefulness and perspectives. Intensive Care Med [Internet].

[21] Akoumianaki E., Maggiore S.M., Valenza F., Bellani G., Jubran A., Loring S.H. (2014 Mar 1). The application of esophageal pressure measurement in patients with respiratory failure. Am J Respir Crit Care Med [Internet].

[22] Calzia E., Lindner K.H., Witt S., Schirmer U., Lange H., Stenz R. (1994). Pressure-time product and work of breathing during biphasic continuous positive airway pressure and assisted spontaneous breathing. Am J Respir Crit Care Med [Internet].

[23] Umbrello M., Formenti P., Lusardi A.C., Guanziroli M., Caccioppola A., Coppola S. (2020 Jul 1). Oesophageal pressure and respiratory muscle ultrasonographic measurements indicate inspiratory effort during pressure support ventilation. Br J Anaesth [Internet].

[24] Bertoni M., Spadaro S., Goligher E.C. (2020 Mar 24). Monitoring patient respiratory effort during mechanical ventilation: lung and diaphragm-protective ventilation. Crit Care [Internet].

[25] Carteaux G., Mancebo J., Mercat A., Dellamonica J., Richard J.C.M., Aguirre-Bermeo H. (2013 Sep). Bedside adjustment of proportional assist ventilation to target a predefined range of respiratory effort. Crit Care Med [Internet].

[26] Marin-Corral J., Dot I., Boguña M., Cecchini L., Zapatero A., Gracia M.P. (2019 Apr 1). Structural differences in the diaphragm of patients following controlled vs assisted and spontaneous mechanical ventilation. Intensive Care Med [Internet].

[27] Goligher E.C., Dres M., Patel B.K., Sahetya S.K., Beitler J.R., Telias I. (2020 Oct 1). Lung- and diaphragm-protective ventilation. Am J Respir Crit Care Med [Internet].

[28] Bello G., Giammatteo V., Bisanti A., Delle Cese L., Rosà T., Menga L.S. (2024 Jun 1). High vs low PEEP in patients with ARDS Exhibiting intense inspiratory effort during assisted ventilation: a randomized crossover trial. Chest [Internet].

[29] Vassilakopoulos T., Petrof B.J. (2004 Feb 1). Ventilator-induced diaphragmatic dysfunction. Am J Respir Crit Care Med [Internet]..

[30] Santana P.V., Cardenas L.Z., Albuquerque A.L.P.de (2023 Mar 1). Diaphragm ultrasound in critically ill patients on mechanical ventilation-evolving concepts. Diagnostics (Basel) [Internet].

[31] Hudson M.B., Smuder A.J., Nelson W.B., Bruells C.S., Levine S., Powers S.K. (2012 Apr). Both high level pressure support ventilation and controlled mechanical ventilation induce diaphragm dysfunction and atrophy. Crit Care Med [Internet].

[32] Peñuelas O., Keough E., López-Rodríguez L., Carriedo D., Gonçalves G., Barreiro E. (2019 Jul 25). Ventilator-induced diaphragm dysfunction: translational mechanisms lead to therapeutical alternatives in the critically ill. Intensive Care Med Exp.

[33] de Vries H.J., Jonkman A.H., de Grooth H.J., Duitman J.W., Girbes A.R.J., Ottenheijm C.A.C. (2022 Feb 1). Lung- and diaphragm-protective ventilation by titrating inspiratory support to diaphragm effort: a randomized clinical trial. Crit Care Med [Internet].

[34] Goligher E.C., Dres M., Fan E., Rubenfeld G.D., Scales D.C., Herridge M.S. (2018 Jan 15). Mechanical ventilation-induced diaphragm atrophy strongly impacts clinical outcomes. Am J Respir Crit Care Med [Internet].

[35] Foti G., Cereda M., Banfi G., Pelosi P., Fumagalli R., Pesenti A. (1997). End-inspiratory airway occlusion: a method to assess the pressure developed by inspiratory muscles in patients with acute lung injury undergoing pressure support. Am J Respir Crit Care Med [Internet].

[36] Jubran A., Van De Graaff W.B., Tobin M.J. (1995). Variability of patient-ventilator interaction with pressure support ventilation in patients with chronic obstructive pulmonary disease. Am J Respir Crit Care Med [Internet].

[37] Natalini G., Buizza B., Granato A., Aniballi E., Pisani L., Ciabatti G. (2021 Aug 1). Non-invasive assessment of respiratory muscle activity during pressure support ventilation: accuracy of end-inspiration occlusion and least square fitting methods. J Clin Monit Comput [Internet].

[38] Gao R., Zhou J.X., Yang Y.L., Xu S.S., Zhou Y.M., Zhang L. (2024). Use of pressure muscle index to predict the contribution of patient’s inspiratory effort during pressure support ventilation: a prospective physiological study. Front Med (Lausanne) [Internet].

[39] Kyogoku M., Shimatani T., Hotz J.C., Newth C.J.L., Bellani G., Takeuchi M. (2021 Mar 1). Direction and magnitude of change in plateau from peak pressure during inspiratory holds can identify the degree of spontaneous effort and elastic workload in ventilated patients. Crit Care Med [Internet].

[40] Bellani G., Grassi A., Sosio S., Gatti S., Kavanagh B.P., Pesenti A. (2019 Sep 1). Driving pressure is associated with outcome during assisted ventilation in acute respiratory distress syndrome. Anesthesiology [Internet].

[41] Bianchi I., Grassi A., Pham T., Telias I., Teggia Droghi M., Vieira F. (2022 Apr 1). Reliability of plateau pressure during patient-triggered assisted ventilation. Analysis of a multicentre database. J Crit Care [Internet].

[42] Yang Y.L., Liu Y., Gao R., Song D.J., Zhou Y.M., Miao M.Y. (2023 Dec 1). Use of airway pressure-based indices to detect high and low inspiratory effort during pressure support ventilation: a diagnostic accuracy study. Ann Intensive Care [Internet].

[43] Docci M., Rezoagli E., Teggia-Droghi M., Coppadoro A., Pozzi M., Grassi A. (2023 Dec 1). Individual response in patient’s effort and driving pressure to variations in assistance during pressure support ventilation. Ann Intensive Care [Internet].

[44] Rudolph M.W., Sietses M., Koopman A.A., Blokpoel R.G.T., Kneyber M.C.J. (2025 Apr 1). Airway occlusion pressure and P0.1 to estimate inspiratory effort and respiratory drive in ventilated children. Pediatr Crit Care Med..

[45] Bertoni M., Telias I., Urner M., Long M., Del Sorbo L., Fan E. (2019 Nov 6). A novel non-invasive method to detect excessively high respiratory effort and dynamic transpulmonary driving pressure during mechanical ventilation. Crit Care [Internet].

[46] De Vries H.J., Tuinman P.R., Jonkman A.H., Liu L., Qiu H., Girbes A.R.J. (2023 Mar 1). Performance of noninvasive airway occlusion maneuvers to assess lung stress and diaphragm effort in mechanically ventilated critically ill patients. Anesthesiology [Internet]..

[47] Albani F., Pisani L., Ciabatti G., Fusina F., Buizza B., Granato A. (2021 Dec 1). Flow Index: a novel, non-invasive, continuous, quantitative method to evaluate patient inspiratory effort during pressure support ventilation. Crit Care [Internet].

[48] Albani F., Fusina F., Ciabatti G., Pisani L., Lippolis V., Franceschetti M.E. (2021 Dec 1). Flow Index accurately identifies breaths with low or high inspiratory effort during pressure support ventilation. Crit Care [Internet].

[49] Miao M.Y., Chen W., Zhou Y.M., Gao R., Song D.J., Wang S.P. (2022 Dec 1). Validation of the flow index to detect low inspiratory effort during pressure support ventilation. Ann Intensive Care [Internet].

[50] Haaksma M.E., Smit J.M., Boussuges A., Demoule A., Dres M., Ferrari G. (2022 Dec 1). EXpert consensus On Diaphragm UltraSonography in the critically ill (EXODUS): a Delphi consensus statement On the measurement of Diaphragm ultrasound-derived parameters in a critical care setting. Crit Care [Internet].

[51] Kim W.Y., Suh H.J., Hong S.B., Koh Y., Lim C.M. (2011 Dec). Diaphragm dysfunction assessed by ultrasonography: influence on weaning from mechanical ventilation. Crit Care Med [Internet].

[52] Pearson S.D., Lin J., Stutz M.R., Lecompte-Osorio P., Pohlman A.S., Wolfe K.S. (2022 Sep 1). Immediate effect of mechanical ventilation mode and sedative infusion on measured diaphragm thickness. Ann Am Thorac Soc [Internet].

[53] Boussuges A., Rives S., Finance J., Chaumet G., Vallée N., Risso J.J. (2021 Oct 27). Ultrasound assessment of diaphragm thickness and thickening: reference values and limits of normality when in a seated position. Front Med (Lausanne) [Internet].

[54] Samanta S., Singh R.K., Baronia A.K., Poddar B., Azim A., Gurjar M. (2017 Nov 13). Diaphragm thickening fraction to predict weaning-a prospective exploratory study. J Intensive Care [Internet].

[55] Urner M., Mitsakakis N., Vorona S., Chen L., Sklar M.C., Dres M. (2021 Apr 1). Identifying subjects at risk for diaphragm atrophy during mechanical ventilation using routinely available clinical data. Respir Care [Internet].

[56] Poddighe D., Van Hollebeke M., Choudhary Y.Q., Campos D.R., Schaeffer M.R., Verbakel J.Y. (2024 Dec 1). Accuracy of respiratory muscle assessments to predict weaning outcomes: a systematic review and comparative meta-analysis. Crit Care [Internet]..

[57] Mahmoodpoor A., Fouladi S., Ramouz A., Shadvar K., Ostadi Z., Soleimanpour H. (2022). Diaphragm ultrasound to predict weaning outcome: systematic review and meta-analysis. Anaesthesiol Intensive Ther [Internet].

[58] Garofalo E., Bruni A., Pelaia C. (2019). Comparisons of two diaphragm ultrasound-teaching programs: a multicenter randomized controlled educational study. Ultrasound J.

[59] Telias I., Junhasavasdikul D., Rittayamai N., Piquilloud L., Chen L., Ferguson N.D. (2020). Airway Occlusion Pressure As an Estimate of Respiratory Drive and Inspiratory Effort during Assisted Ventilation. Am J Respir Crit Care Med [Internet].

[60] Van Oosten J.P., Akoumianaki E., Jonkman A.H. (2025 Feb 1). Monitoring respiratory muscles effort during mechanical ventilation. Curr Opin Crit Care..

[61] Lescroart M., Blanchard F., Constantin J.M., Specklin M., Revol A., Hani H. (2025 May). Lung resistance - but not compliance - impairs P0.1 and maximal inspiratory pressure measurements. Anaesth Crit Care Pain Med..

[62] Smits F.E., Rietveld P.J., Snoep J.W.M., van der Velde-Quist F. (2025 Aug 1). P0.1 is an unreliable measure of effort in support mechanical ventilation in comparison with esophageal-derived measures of effort: a comparison study. Crit Care Med..

[63] Rittayamai N., Beloncle F., Goligher E.C., Chen L., Mancebo J., Richard J.C.M. (2017 Dec 1). Effect of inspiratory synchronization during pressure-controlled ventilation on lung distension and inspiratory effort. Ann Intensive Care [Internet].

[64] Le Marec J., Hajage D., Decavèle M., Schmidt M., Laurent I., Ricard J.D. (2024 Jul 15). High airway occlusion pressure is associated with dyspnea and increased mortality in critically ill mechanically ventilated patients. Am J Respir Crit Care Med [Internet].

[65] Pletsch-Assuncao R., Pereira M.C., Ferreira J.G., Cardenas L.Z., De Albuquerque A.L.P., De Carvalho C.R.R. (2018). Accuracy of invasive and noninvasive parameters for diagnosing ventilatory overassistance during pressure support ventilation. Crit Care Med [Internet].

[66] Vargas F., Boyer A., Bui H.N., Salmi L.R., Guenard H., Gruson D. (2008 Dec). Respiratory failure in chronic obstructive pulmonary disease after extubation: value of expiratory flow limitation and airway occlusion pressure after 0.1 second (P0.1). J Crit Care [Internet].

[67] Beloncle F., Piquilloud L., Olivier P.Y., Vuillermoz A., Yvin E., Mercat A. (2019 Dec 1). Accuracy of P0.1 measurements performed by ICU ventilators: a bench study. Ann Intensive Care [Internet].

[68] Tonelli R., Protti A., Spinelli E., Grieco D.L., Yoshida T., Jonkman A.H. (2025 Jul 31). Assessing inspiratory drive and effort in critically ill patients at the bedside. Crit Care..

